# Crystal structure of 4-chloro-2-{(*E*)-[(3,4-di­methyl­phen­yl)imino]­meth­yl}phenol

**DOI:** 10.1107/S2056989015009354

**Published:** 2015-05-23

**Authors:** Muhammad Salim, Muhammad Nawaz Tahir, Munawar Ali Munawar, Muhammad Shahid, Hazoor Ahmad Shad

**Affiliations:** a Department of Chemistry, University of the Punjab, Lahore, Punjab, Pakistan; b Department of Physics, University of Sargodha, Sargodha, Punjab, Pakistan; c Department of Chemistry, University of Sargodha, Sargodha, Punjab, Pakistan

**Keywords:** crystal structure, phenol, intra­molecular hydrogen bonding, C—H⋯π inter­actions

## Abstract

In the title compound, C_15_H_14_ClNO, which is isostructural with its bromo analogue [Tahir *et al.* (2012 ▸). *Acta Cryst.*, E**68**, o2730], the dihedral angle between the planes of the aromatic rings is 2.71 (7)° and an intra­molecular O—H⋯N hydrogen bond closes an *S*(6) ring. In the crystal, extremely weak C—H⋯π inter­actions link the mol­ecules into a three-dimensional network.

## Related literature   

For related structures, see: Demircioğlu *et al.* (2014 ▸); Jin *et al.* (2012 ▸); Sun *et al.* (2013 ▸); Tahir *et al.* (2012 ▸).
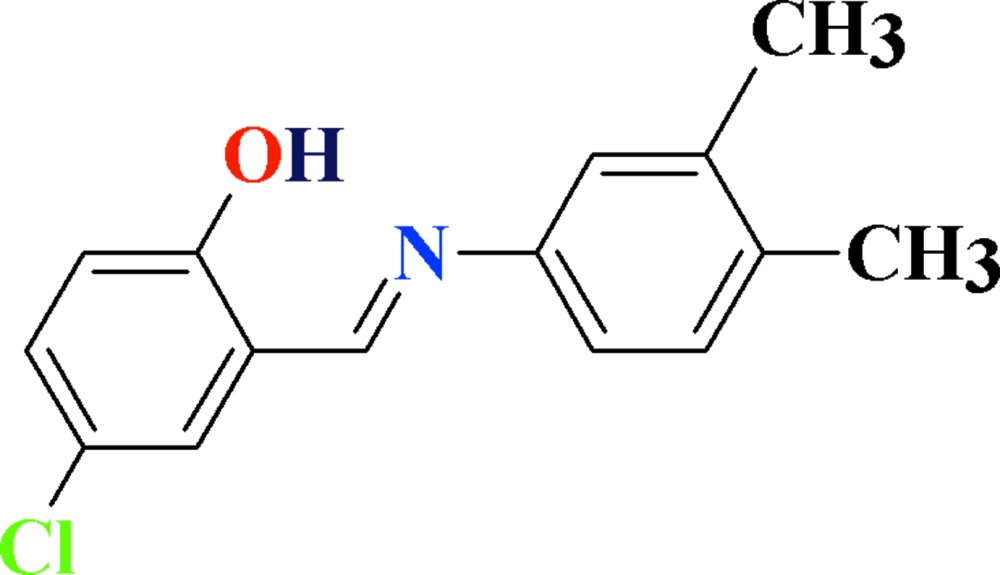


## Experimental   

### Crystal data  

C_15_H_14_ClNO
*M* *_r_* = 259.72
Monoclinic, 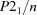

*a* = 12.1875 (10) Å
*b* = 7.4438 (5) Å
*c* = 14.3141 (12) Å
β = 101.549 (4)°
*V* = 1272.30 (17) Å^3^
*Z* = 4
Mo *K*α radiation
μ = 0.29 mm^−1^
*T* = 296 K
0.25 × 0.20 × 0.14 mm

### Data collection  

Bruker Kappa APEXII CCD diffractometer
Absorption correction: multi-scan (*SADABS*; Bruker, 2007 ▸) *T*_min_ = 0.933, *T*_max_ = 0.968
10293 measured reflections
2785 independent reflections
1871 reflections with *I* > 2σ(*I*)
*R*_int_ = 0.024

### Refinement  

*R*[*F* ^2^ > 2σ(*F* ^2^)] = 0.041
*wR*(*F* ^2^) = 0.116
*S* = 1.04
2785 reflections
166 parameters
H-atom parameters constrained
Δρ_max_ = 0.24 e Å^−3^
Δρ_min_ = −0.22 e Å^−3^

### 

Data collection: *APEX2* (Bruker, 2007 ▸); cell refinement: *SAINT* (Bruker, 2007 ▸); data reduction: *SAINT*; program(s) used to solve structure: *SHELXS97* (Sheldrick, 2008 ▸); program(s) used to refine structure: *SHELXL2014* (Sheldrick, 2015 ▸); molecular graphics: *ORTEP-3 for Windows* (Farrugia, 2012 ▸) and *PLATON* (Spek, 2009 ▸); software used to prepare material for publication: *WinGX* (Farrugia, 2012 ▸) and *PLATON*.

## Supplementary Material

Crystal structure: contains datablock(s) global, I. DOI: 10.1107/S2056989015009354/hb7424sup1.cif

Structure factors: contains datablock(s) I. DOI: 10.1107/S2056989015009354/hb7424Isup2.hkl

Supporting information file. DOI: 10.1107/S2056989015009354/hb7424Isup3.cml

**Fig. 1.**
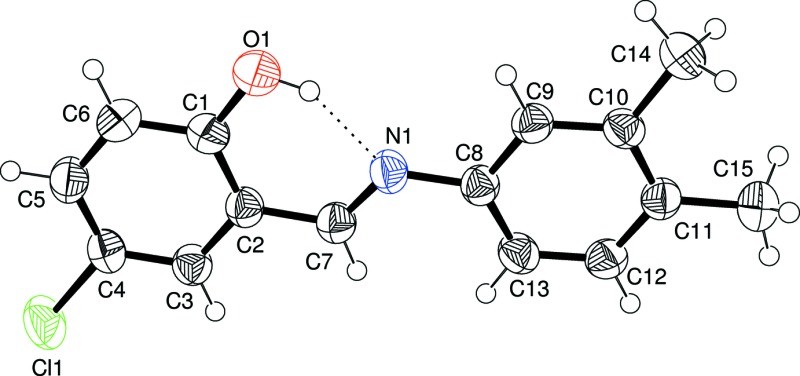
View of the title compound with displacement ellipsoids drawn at the 50% probability level. The dotted line shows intramolecular H-bonding.

. DOI: 10.1107/S2056989015009354/hb7424fig1.tif
View of the title compound with displacement ellipsoids drawn at the 50% probability level. The dotted line shows intra­molecular H-bonding.

**Fig. 2.**
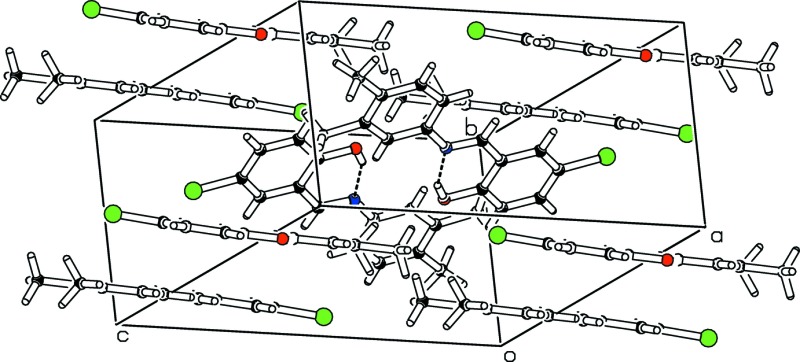
Packing diagram for the title compound.

. DOI: 10.1107/S2056989015009354/hb7424fig2.tif
Packing diagram for the title compound.

CCDC reference: 1401503

Additional supporting information:  crystallographic information; 3D view; checkCIF report

## Figures and Tables

**Table 1:** Hydrogen-bond geometry (, ) *Cg*1 and *Cg*2 are the centroids of the C1C6 and C8C13 benzene rings, respectively.

*D*H*A*	*D*H	H*A*	*D* *A*	*D*H*A*
O1H1N1	0.82	1.87	2.5998(19)	149
C3H3*Cg*1^i^	0.93	2.98	3.732(2)	139
C6H6*Cg*2^ii^	0.93	2.93	3.576(2)	128
C14H14*B* *Cg*2^iii^	0.96	2.96	3.656(2)	131

Symmetry codes: (i) 
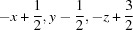
; (ii) 
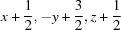
; (iii) 
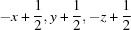
.

## supplementary crystallographic information

### S1. Comment

The title compound, (I, Fig. 1) has been synthesized in continuation of forming different derivatives of 3,4-dimethylaniline. (I) will also be utilized for synthesizing different metal complexes.

The crystal structures of 4-bromo-2-((*E*)-[(3,4-dimethylphenyl)imino]methyl)phenol (Tahir *et al.*, 2012), 2-((3,4-dimethylphenyl)carbonoimidoyl)-3-methoxyphenol (Demircioğlu *et al.*, 2014), *N*-[(*E*)-4-bromobenzylidene]-3,4-dimethylaniline (Sun *et al.*, 2013) and *N*-[(*E*)-4-fluorobenzylidene]-3,4-dimethylaniline (Jin *et al.*, 2012) have been published which are related to the title compound.

The title compound is isostructural to 4-bromo-2-((*E*)-[(3,4-dimethyl phenyl)imino]methyl)phenol (Tahir *et al.*, 2012) and is almost planar with r. m. s. deviation of 0.0325 Å, with maximum deviation of 0.0803 (9) Å for Cl1 atom from the mean square plane. There exist intramolecular H-bonding of O—H···N type (Table 1, Fig. 1) with *S*(6) ring motif. There exist C—H···π interactions (Table 1).

### S2. Experimental

Equimolar quantities of 5-chlorosalicylaldehyde and 3,4-dimethylaniline were refluxed in methanol for 3 h. The solution was kept at room temperature for crystallization which affoarded light yellow plates after 72 h.

### S3. Refinement

The H atoms were positioned geometrically (C–H = 0.93–0.96 Å, O—H= 0.82 Å) and refined as riding with *U*_iso_(H) = *xU*_eq_(C, O), where *x* = 1.5 for methyl & hydroxy and *x* = 1.2 for other H-atoms.

### Crystal data

**Table:**

C_15_H_14_ClNO	*F*(000) = 544
*M**_r_* = 259.72	*D*_x_ = 1.356 Mg m^−^^3^
Monoclinic, *P*2_1_/*n*	Mo *K*α radiation, λ = 0.71073 Å
*a* = 12.1875 (10) Å	Cell parameters from 1871 reflections
*b* = 7.4438 (5) Å	θ = 2.0–27.0°
*c* = 14.3141 (12) Å	µ = 0.29 mm^−^^1^
β = 101.549 (4)°	*T* = 296 K
*V* = 1272.30 (17) Å^3^	Plate, light yellow
*Z* = 4	0.25 × 0.20 × 0.14 mm

### Data collection

**Table:**

Bruker Kappa APEXII CCD diffractometer	2785 independent reflections
Radiation source: fine-focus sealed tube	1871 reflections with *I* > 2σ(*I*)
Graphite monochromator	*R*_int_ = 0.024
Detector resolution: 7.80 pixels mm^-1^	θ_max_ = 27.0°, θ_min_ = 2.0°
ω scans	*h* = −15→12
Absorption correction: multi-scan (*SADABS*; Bruker, 2007)	*k* = −8→9
*T*_min_ = 0.933, *T*_max_ = 0.968	*l* = −18→15
10293 measured reflections	

### Refinement

**Table:**

Refinement on *F*^2^	Primary atom site location: structure-invariant direct methods
Least-squares matrix: full	Secondary atom site location: difference Fourier map
*R*[*F*^2^ > 2σ(*F*^2^)] = 0.041	Hydrogen site location: inferred from neighbouring sites
*wR*(*F*^2^) = 0.116	H-atom parameters constrained
*S* = 1.04	*w* = 1/[σ^2^(*F*_o_^2^) + (0.0519*P*)^2^ + 0.2053*P*] where *P* = (*F*_o_^2^ + 2*F*_c_^2^)/3
2785 reflections	(Δ/σ)_max_ = 0.001
166 parameters	Δρ_max_ = 0.24 e Å^−^^3^
0 restraints	Δρ_min_ = −0.22 e Å^−^^3^

### Special details

**Table:**

Geometry. All e.s.d.'s (except the e.s.d. in the dihedral angle between two l.s. planes) are estimated using the full covariance matrix. The cell e.s.d.'s are taken into account individually in the estimation of e.s.d.'s in distances, angles and torsion angles; correlations between e.s.d.'s in cell parameters are only used when they are defined by crystal symmetry. An approximate (isotropic) treatment of cell e.s.d.'s is used for estimating e.s.d.'s involving l.s. planes.
Refinement. Refinement of *F*^2^ against ALL reflections. The weighted *R*-factor *wR* and goodness of fit *S* are based on *F*^2^, conventional *R*-factors *R* are based on *F*, with *F* set to zero for negative *F*^2^. The threshold expression of *F*^2^ > σ(*F*^2^) is used only for calculating *R*-factors(gt) *etc*. and is not relevant to the choice of reflections for refinement. *R*-factors based on *F*^2^ are statistically about twice as large as those based on *F*, and *R*-factors based on ALL data will be even larger.

### Fractional atomic coordinates and isotropic or equivalent isotropic displacement parameters (Å^2^)

**Table:**

	*x*	*y*	*z*	*U*_iso_*/*U*_eq_	
Cl1	0.31558 (4)	0.47763 (8)	0.94032 (3)	0.0702 (2)	
O1	0.45252 (11)	0.6419 (2)	0.58212 (9)	0.0684 (4)	
H1	0.4036	0.6151	0.5361	0.103*	
N1	0.26240 (12)	0.51118 (17)	0.49303 (10)	0.0442 (4)	
C1	0.41674 (14)	0.6064 (2)	0.66249 (12)	0.0462 (4)	
C2	0.31233 (14)	0.5273 (2)	0.66205 (11)	0.0400 (4)	
C3	0.28167 (14)	0.4894 (2)	0.74847 (12)	0.0434 (4)	
H3	0.2128	0.4363	0.7492	0.052*	
C4	0.35292 (14)	0.5305 (2)	0.83271 (12)	0.0447 (4)	
C5	0.45476 (15)	0.6104 (2)	0.83316 (12)	0.0499 (4)	
H5	0.5022	0.6383	0.8907	0.060*	
C6	0.48620 (15)	0.6486 (2)	0.74880 (13)	0.0524 (5)	
H6	0.5549	0.7035	0.7493	0.063*	
C7	0.23682 (15)	0.4821 (2)	0.57324 (12)	0.0432 (4)	
H7	0.1679	0.4304	0.5753	0.052*	
C8	0.18970 (13)	0.4694 (2)	0.40504 (11)	0.0394 (4)	
C9	0.23021 (14)	0.5050 (2)	0.32363 (12)	0.0414 (4)	
H9	0.3016	0.5534	0.3296	0.050*	
C10	0.16807 (14)	0.4712 (2)	0.23319 (12)	0.0407 (4)	
C11	0.06064 (14)	0.3993 (2)	0.22426 (12)	0.0429 (4)	
C12	0.02122 (14)	0.3625 (2)	0.30650 (12)	0.0455 (4)	
H12	−0.0499	0.3133	0.3010	0.055*	
C13	0.08330 (14)	0.3962 (2)	0.39585 (12)	0.0456 (4)	
H13	0.0544	0.3701	0.4497	0.055*	
C14	0.21523 (18)	0.5150 (3)	0.14640 (13)	0.0576 (5)	
H14A	0.2165	0.4083	0.1089	0.086*	
H14B	0.2901	0.5603	0.1659	0.086*	
H14C	0.1693	0.6042	0.1090	0.086*	
C15	−0.01190 (16)	0.3653 (3)	0.12815 (13)	0.0599 (5)	
H15A	0.0236	0.2788	0.0943	0.090*	
H15B	−0.0225	0.4756	0.0927	0.090*	
H15C	−0.0832	0.3200	0.1360	0.090*	

### Atomic displacement parameters (Å^2^)

**Table:**

	*U*^11^	*U*^22^	*U*^33^	*U*^12^	*U*^13^	*U*^23^
Cl1	0.0742 (4)	0.1002 (4)	0.0366 (3)	−0.0049 (3)	0.0120 (2)	0.0047 (2)
O1	0.0658 (9)	0.0930 (10)	0.0483 (8)	−0.0276 (8)	0.0159 (7)	0.0033 (7)
N1	0.0472 (8)	0.0498 (8)	0.0349 (8)	−0.0009 (6)	0.0069 (6)	−0.0023 (6)
C1	0.0493 (10)	0.0475 (10)	0.0428 (10)	−0.0042 (8)	0.0116 (8)	0.0025 (7)
C2	0.0427 (9)	0.0390 (9)	0.0376 (9)	0.0015 (7)	0.0061 (7)	−0.0012 (7)
C3	0.0430 (10)	0.0469 (10)	0.0407 (9)	0.0001 (7)	0.0093 (8)	−0.0005 (7)
C4	0.0493 (11)	0.0476 (10)	0.0364 (9)	0.0048 (8)	0.0065 (8)	0.0002 (7)
C5	0.0519 (11)	0.0500 (10)	0.0432 (10)	−0.0005 (8)	−0.0013 (8)	−0.0027 (8)
C6	0.0476 (11)	0.0523 (11)	0.0550 (12)	−0.0103 (8)	0.0048 (9)	−0.0008 (9)
C7	0.0440 (10)	0.0450 (9)	0.0404 (10)	−0.0016 (7)	0.0080 (8)	−0.0015 (7)
C8	0.0421 (10)	0.0383 (9)	0.0376 (9)	0.0011 (7)	0.0073 (7)	−0.0016 (7)
C9	0.0404 (9)	0.0421 (9)	0.0423 (10)	−0.0036 (7)	0.0102 (7)	−0.0015 (7)
C10	0.0480 (10)	0.0368 (9)	0.0389 (9)	0.0015 (7)	0.0125 (7)	0.0007 (7)
C11	0.0482 (10)	0.0368 (9)	0.0417 (10)	0.0013 (7)	0.0040 (7)	−0.0007 (7)
C12	0.0410 (9)	0.0474 (10)	0.0482 (10)	−0.0044 (8)	0.0091 (8)	0.0017 (8)
C13	0.0467 (10)	0.0520 (10)	0.0400 (10)	−0.0016 (8)	0.0132 (8)	0.0029 (8)
C14	0.0672 (13)	0.0661 (12)	0.0422 (10)	−0.0091 (9)	0.0171 (9)	0.0008 (8)
C15	0.0621 (12)	0.0674 (13)	0.0461 (11)	−0.0079 (10)	0.0008 (9)	−0.0017 (9)

### Geometric parameters (Å, º)

**Table:**

Cl1—C4	1.7365 (17)	C8—C13	1.389 (2)
O1—C1	1.336 (2)	C9—C10	1.386 (2)
O1—H1	0.8200	C9—H9	0.9300
N1—C7	1.267 (2)	C10—C11	1.396 (2)
N1—C8	1.422 (2)	C10—C14	1.505 (2)
C1—C6	1.387 (2)	C11—C12	1.385 (2)
C1—C2	1.401 (2)	C11—C15	1.500 (2)
C2—C3	1.391 (2)	C12—C13	1.372 (2)
C2—C7	1.452 (2)	C12—H12	0.9300
C3—C4	1.372 (2)	C13—H13	0.9300
C3—H3	0.9300	C14—H14A	0.9600
C4—C5	1.375 (2)	C14—H14B	0.9600
C5—C6	1.368 (2)	C14—H14C	0.9600
C5—H5	0.9300	C15—H15A	0.9600
C6—H6	0.9300	C15—H15B	0.9600
C7—H7	0.9300	C15—H15C	0.9600
C8—C9	1.379 (2)		
			
C1—O1—H1	109.5	C8—C9—H9	118.9
C7—N1—C8	122.85 (15)	C10—C9—H9	118.9
O1—C1—C6	118.44 (15)	C9—C10—C11	118.83 (15)
O1—C1—C2	122.14 (15)	C9—C10—C14	120.27 (16)
C6—C1—C2	119.42 (15)	C11—C10—C14	120.89 (15)
C3—C2—C1	119.12 (15)	C12—C11—C10	118.45 (15)
C3—C2—C7	119.72 (15)	C12—C11—C15	120.34 (16)
C1—C2—C7	121.15 (15)	C10—C11—C15	121.20 (16)
C4—C3—C2	120.07 (16)	C13—C12—C11	122.41 (16)
C4—C3—H3	120.0	C13—C12—H12	118.8
C2—C3—H3	120.0	C11—C12—H12	118.8
C3—C4—C5	120.82 (16)	C12—C13—C8	119.34 (15)
C3—C4—Cl1	119.80 (14)	C12—C13—H13	120.3
C5—C4—Cl1	119.37 (13)	C8—C13—H13	120.3
C6—C5—C4	119.86 (16)	C10—C14—H14A	109.5
C6—C5—H5	120.1	C10—C14—H14B	109.5
C4—C5—H5	120.1	H14A—C14—H14B	109.5
C5—C6—C1	120.69 (16)	C10—C14—H14C	109.5
C5—C6—H6	119.7	H14A—C14—H14C	109.5
C1—C6—H6	119.7	H14B—C14—H14C	109.5
N1—C7—C2	121.73 (16)	C11—C15—H15A	109.5
N1—C7—H7	119.1	C11—C15—H15B	109.5
C2—C7—H7	119.1	H15A—C15—H15B	109.5
C9—C8—C13	118.75 (15)	C11—C15—H15C	109.5
C9—C8—N1	116.19 (14)	H15A—C15—H15C	109.5
C13—C8—N1	125.06 (14)	H15B—C15—H15C	109.5
C8—C9—C10	122.23 (15)		
			
O1—C1—C2—C3	178.21 (15)	C7—N1—C8—C9	−178.57 (14)
C6—C1—C2—C3	−1.3 (2)	C7—N1—C8—C13	1.2 (3)
O1—C1—C2—C7	−0.9 (3)	C13—C8—C9—C10	0.4 (2)
C6—C1—C2—C7	179.60 (15)	N1—C8—C9—C10	−179.85 (13)
C1—C2—C3—C4	0.4 (2)	C8—C9—C10—C11	0.3 (2)
C7—C2—C3—C4	179.50 (14)	C8—C9—C10—C14	179.07 (14)
C2—C3—C4—C5	0.5 (3)	C9—C10—C11—C12	−0.8 (2)
C2—C3—C4—Cl1	−178.37 (12)	C14—C10—C11—C12	−179.62 (15)
C3—C4—C5—C6	−0.4 (3)	C9—C10—C11—C15	178.03 (15)
Cl1—C4—C5—C6	178.42 (13)	C14—C10—C11—C15	−0.7 (2)
C4—C5—C6—C1	−0.5 (3)	C10—C11—C12—C13	0.8 (3)
O1—C1—C6—C5	−178.16 (16)	C15—C11—C12—C13	−178.10 (15)
C2—C1—C6—C5	1.4 (3)	C11—C12—C13—C8	−0.1 (3)
C8—N1—C7—C2	−179.54 (13)	C9—C8—C13—C12	−0.5 (2)
C3—C2—C7—N1	−178.66 (14)	N1—C8—C13—C12	179.79 (15)
C1—C2—C7—N1	0.4 (3)		

### Hydrogen-bond geometry (Å, º)

Cg1 and Cg2 are the centroids of the C1–C6 and C8–C13 benzene rings, respectively.

**Table:**

*D*—H···*A*	*D*—H	H···*A*	*D*···*A*	*D*—H···*A*
O1—H1···N1	0.82	1.87	2.5998 (19)	149
C3—H3···*Cg*1^i^	0.93	2.98	3.732 (2)	139
C6—H6···*Cg*2^ii^	0.93	2.93	3.576 (2)	128
C14—H14*B*···*Cg*2^iii^	0.96	2.96	3.656 (2)	131

Symmetry codes: (i) −*x*+1/2, *y*−1/2, −*z*+3/2; (ii) *x*+1/2, −*y*+3/2, *z*+1/2; (iii) −*x*+1/2, *y*+1/2, −*z*+1/2.
