# Supplementary figures and images for: Crystal structure of 4-chloro-2-{(E)-[(3,4-di­methyl­phen­yl)imino]­meth­yl}phenol
Source: Acta Crystallogr E Crystallogr Commun. 2015 May 23;71(Pt 6):o416. doi: 10.1107/S2056989015009354 (PMC4459303; doi:10.1107/S2056989015009354)

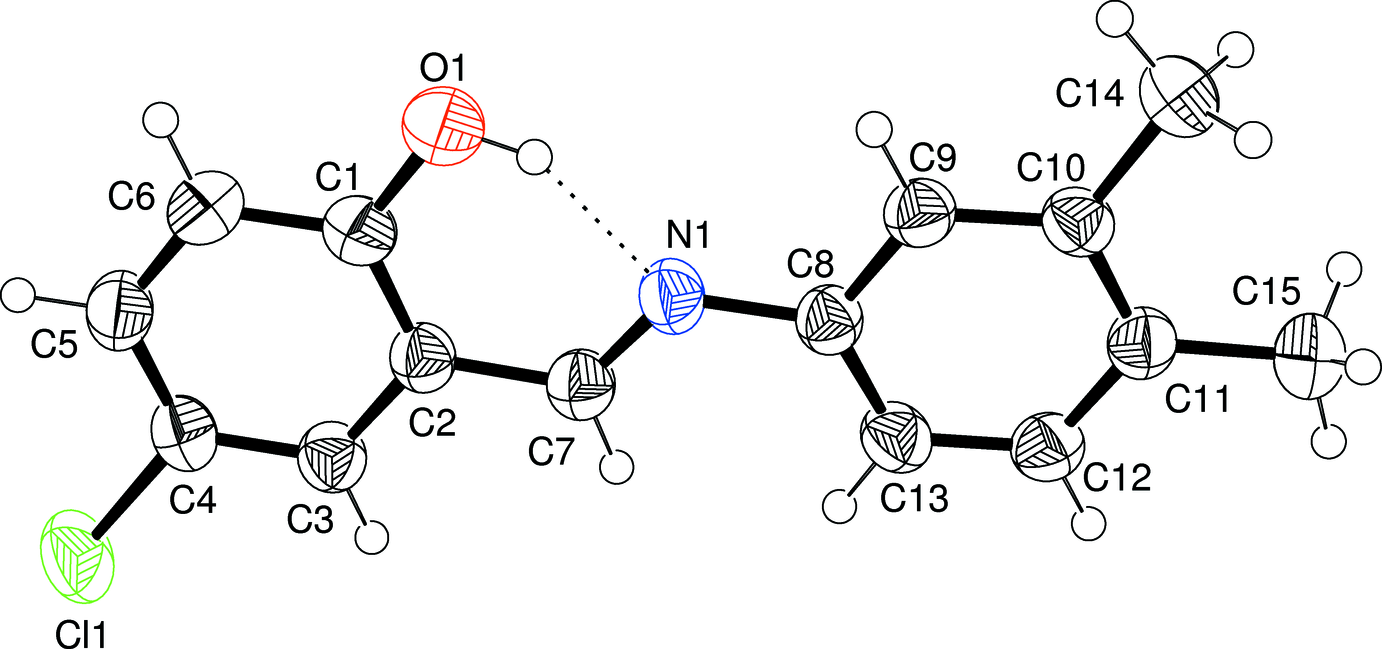

Supplement: Supplementary file 4 [file e-71-0o416-fig1.tif]

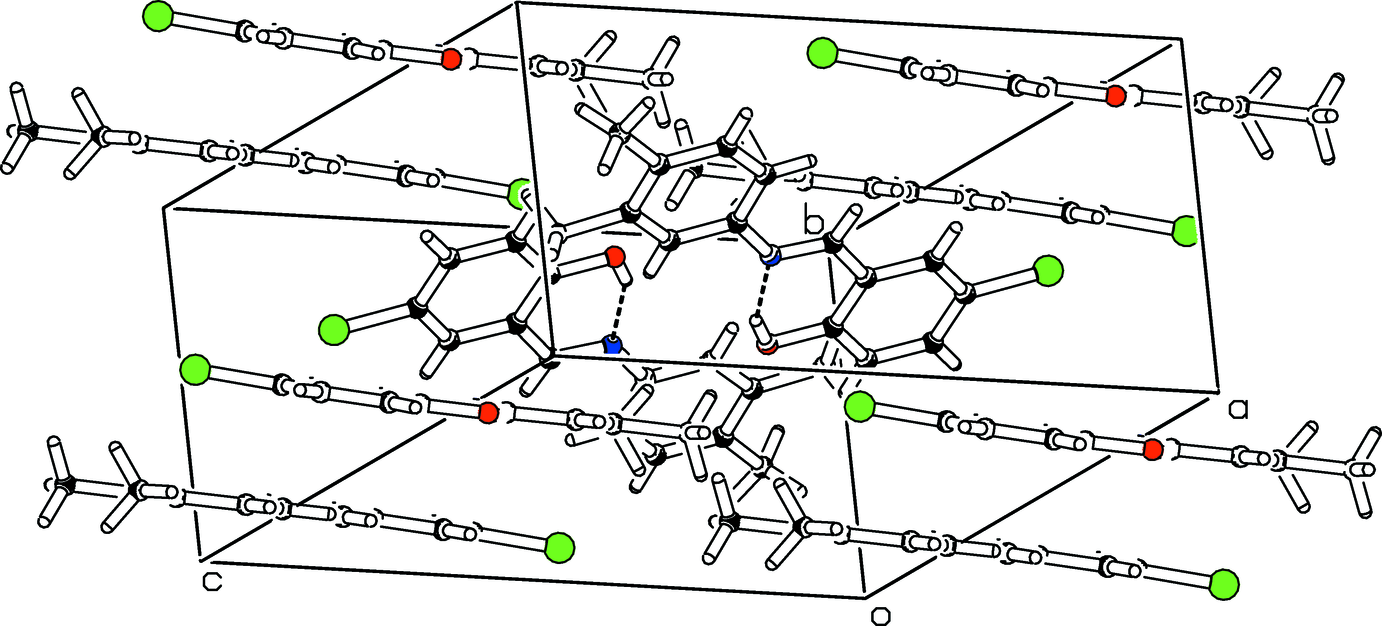

Supplement: Supplementary file 5 [file e-71-0o416-fig2.tif]
